# The Kinase Inhibitor GNF-7 Is Synthetically Lethal in Topoisomerase 1-Deficient Ewing Sarcoma

**DOI:** 10.3390/cancers17152475

**Published:** 2025-07-26

**Authors:** Carly M. Sayers, Morgan B. Carter, Haiyan Lei, Arnulfo Mendoza, Steven Shema, Xiaohu Zhang, Kelli Wilson, Lu Chen, Carleen Klumpp-Thomas, Craig J. Thomas, Christine M. Heske, Jack F. Shern

**Affiliations:** 1 Pediatric Oncology Branch, Center for Cancer Research, National Cancer Institute, National Institutes of Health, Bethesda, MD 20892, USA; carly.sayers@nih.gov (C.M.S.);; 2 CCR Genomics Core, Center for Cancer Research, National Cancer Institute, National Institutes of Health, Bethesda, MD 20892, USA; 3 Division of Preclinical Innovation, National Center for Advancing Translational Sciences, National Institutes of Health, Rockville, MD 20850, USA

**Keywords:** Ewing sarcoma, topoisomerase, irinotecan, kinase inhibitor, GNF-7, focal adhesion

## Abstract

Background/Objectives: Ewing sarcoma (ES), a highly aggressive bone and soft tissue cancer occurring in children and young adults, is defined by the ETS fusion oncoprotein EWS::FLI1. Although event-free survival rates remain high in ES patients with localized disease, those with metastatic or relapsed disease face poor long-term survival odds. Topoisomerase 1 (TOP1) inhibitors are commonly used therapeutics in ES relapse regimens. Methods: In this work, we used a genome-wide CRISPR knockout library screen to identify the deletion of the *TOP1* gene as a mechanism for resistance to topoisomerase 1 inhibitors. Using isogenic cell line models, we performed a high-throughput small-molecule screen to discover a small molecule, GNF-7, which had an IC50 that was 10-fold lower in *TOP1*-deficient cells when compared to the wild-type cells. Results: The characterization of GNF-7 demonstrated the molecule was highly active in the inhibition of CSK, p38α, EphA2, Lyn, and ZAK and specifically downregulated genes induced by the EWS::FLI1 fusion oncoprotein. Conclusions: Together, these results suggest that GNF-7 or small molecules with a similar kinase profile could be effective treatments for ES patients in combination with TOP1 inhibitors or for those patients who have developed resistance to TOP1 inhibitors.

## Simple Summary

Using a comprehensive genetic screening strategy coupled with high-throughput small-molecule screening, we discovered the multi-kinase inhibitor GNF-7 had increased potency in *TOP1*-deficient Ewing sarcoma cells. The kinase profiling of GNF-7 demonstrated broad activity with inhibition of CSK, p38α, EphA2, Lyn, and ZAK.

## 1. Introduction

Refractory disease after therapy is a fundamental challenge to the treatment of sarcomas. Ewing sarcoma (ES), characterized by the *EWS::FLI1* fusion oncogene, is the second most common bone tumor in children. ES is treated with aggressive multimodal therapy including chemotherapy, radiotherapy, and surgery, resulting in initial tumor responses in the majority of patients [1]. Unfortunately, despite these intensive therapeutic regimens, survival for patients with recurrent disease remains poor [2]. Therefore, the selection of novel therapeutic agents and drug combinations designed to target therapeutically resistant cells is needed to improve survival for these patients.

Potential salvage therapies for ES frequently employ camptothecin (CPT) derivative Topoisomerase 1 inhibitors (TOP1i), such as Topotecan or Irinotecan [3]. TOP1 plays a critical role in DNA transcription and replication by catalyzing a single-strand nick in the DNA, thereby allowing a supercoiled double helix to relax [4]. Normally, TOP1 re-ligates the nicked DNA after it unwinds, keeping the structure intact [4]. However, in the presence of TOP1i, the re-ligation of the nicked DNA is impaired [5,6]. When transcription or replication complexes encounter these genomic loci, DNA double strand breaks are formed, the accumulation of which can ultimately lead to cell death [5]. It has been previously shown that the intrinsic sensitivity of TOP1 to camptothecin is dependent on the composition of the core domain and the C terminus of the protein [7]. Despite impressive initial responses in the clinic with TOP1i regimens, the majority of patients will ultimately develop TOP1i resistance and disease progression [8]. It is not surprising, therefore, that a large research focus has been placed on understanding the mechanisms of cancer cell resistance to TOP1i. Elegant studies have documented a number of mechanisms utilized by tumor cells to become resistant to TOP1 inhibitors, such as upregulation or mutation of the proteins involved in drug efflux and mutations in the TOP1 protein [9,10,11,12,13]. Notably, a potential mechanism of resistance to TOP1 inhibitors is the downregulation of TOP1 protein expression [14,15,16,17]. While the mechanisms of resistance are characterized, unbiased small-molecule screening of TOP1-deficient cells remains relatively unexplored.

In this study, we sought to identify the primary mechanism of ES cell resistance to TOP1i and discover small molecules that would be selectively toxic to TOP1i resistant cells. To do this, we performed a series of genetic and small-molecule high-throughput screens designed to identify the mechanism of resistance as well as lead compounds that would be selectively toxic to the resistant tumor cell. This work discovered a tyrosine kinase inhibitor, GNF-7, that has previously been characterized as a Type II kinase inhibitor and has been developed to inhibit multidrug-resistant mutant BCR-ABL [18]. Extensive downstream analysis of GNF-7 demonstrated a unique multi-tyrosine kinase-inhibitory pharmacology that was responsible for the observed synthetic lethality with *TOP1* gene loss.

## 2. Materials and Methods

### 2.1. Cell Culture

The ES cell lines EW8, TC71, and RDES were kindly provided by Dr. Lee Helman (National Cancer Institute (NCI), National Institutes of Health (NIH)) and were grown in RPMI-1640 (Thermo Fisher Scientific, Waltham, MA, USA) media supplemented with 10% FBS and 100 U/mL penicillin/100 μg/mL streptomycin/29.2 μg/mL L-glutamine (Thermo Fisher Scientific). These Ewing sarcoma cell lines have been genomically and transcriptomically characterized previously [19]. Lenti-X HEK293T cells were originally purchased from Takara Bio and kindly gifted by Dr. Carol Thiele (Pediatric Oncology Branch, NCI). These cells were maintained in DMEM media supplemented with 10% FBS and 100 U/mL penicillin/100 μg/mL streptomycin/29.2 μg/mL L-glutamine. All cell lines were verified by STR profiling and were routinely tested for mycoplasma. Once thawed, cells were cultured for no more than 15 passages. Finally, all cells were grown in an incubator at 37 °C with 5% CO_2_.

### 2.2. Genome-Scale CRISPR-Cas9 Knockout (GeCKO) Library Screen

The human CRISPR knockout pooled library v2 was a gift from Feng Zhang (Addgene, Watertown, MA, USA), and lentivirus production and cell line screening were performed according to the GeCKO knockout screening protocol published by the Zhang lab [20,21]. Briefly, GeCKO 1-vector library A and B plasmids were packaged into Lenti-X HEK293T cells, and the titer of the resulting lentiviral libraries was determined using CellTiter-Glo^®^ (Promega, Madison, WI, USA), with the luminescent readout being measured with the SpectraMax M3 plate reader (Molecular Devices, San Jose, CA, USA). EW8 cells were screened two separate times using both library A and library B virus pools for a total of four biological replicates. For the screens, EW8 cells were plated onto flasks (1.1 × 10^8^ cells for library A and 9.7 × 10^7^ cells for library B) then infected 24 h later with the GeCKO library virus at an MOI of 0.3. Transduced EW8 cells were selected for 10 days with 0.25 μg/mL puromycin (Gibco, Frederick, MD, USA) starting 24 h after infection, during which time cells were passaged every 3–4 days. After selection, the cells were plated into two experimental groups (minimum of 3.3 × 10^7^ or 2.9 × 10^7^ cells per group for library A or library B, respectively), and treatment occurred 24 h after plating. One group of cells was treated with 8 nM camptothecin (CPT) (Selleckchem, Houston, TX, USA), a dose which is sufficient to kill all normal EW8 cells, while the other group was treated with an equal volume of DMSO (Millipore Sigma, Burlington, MA, USA) as a vehicle control. Cells were passaged, or media was changed and fresh treatment was added, every 3–4 days. After 27 days of continuous treatment, 2.5 × 10^7^ cells were collected from each treatment group. Genomic DNA was extracted from the cell pellets using the Quick gDNA MidiPrep kit from Zymo Research (Irvine, CA, USA). PCR was performed on the extracted gDNA according to the published GeCKO protocol using KAPA HiFi HotStart ReadyMix (Kapa Biosystems, Wilmington, MA, USA) and the published primer sequences. The PCR product was purified using Zymo-Spin V columns with reservoirs, DNA Wash Buffer, DNA Binding Buffer, and DNA Elution Buffer (Zymo Research). Purified PCR product was electrophoresed on a 2% agarose gel, and the 270 bp bands were extracted using the QIAquick Gel Extraction kit (Qiagen, Germantown, MD, USA) according to the manufacturer’s instructions. The final product was sequenced by the NCI CCR Genomics Core (Bethesda, MD) using the high-output NextSeq platform (Illumina, San Diego, CA, USA). Sample comparisons were made using the MAGeCK algorithm [22].

### 2.3. Lentiviral Infection

To generate ES cells with knockout of *TOP1*, an sgRNA targeting *TOP1* exon 3 was cloned into the pLentiCRISPR v2 backbone, which also contained the gene for Cas9 (GenScript, Piscataway, NJ, USA). Lentivirus was produced by cotransfecting Lenti-X HEK293T cells (Takara Bio, San Jose, CA, USA) with the packaging vectors psPAX2 and pMD2.G (kindly gifted by Dr. Carol Thiele) using Lipofectamine 2000 (Thermo Fisher). Cells were transduced at an MOI of 0.5, and infected cells were selected using either 0.25 μg/mL puromycin (EW8) or by fluorescence-activated cell sorting for cells expressing GFP (TC71 and RDES) using the LSRFortessa (BD Biosciences, San Jose, CA, USA). Individual clones were isolated by sparsely seeding cells onto plates, allowing the cells to grow until colonies were visible, then selecting isolated colonies using sterile cloning disks (Bel-Art, Wayne, NJ, USA) and transferring the discs to individual wells of a 24-well plate containing media. Once cells had expanded, total protein lysates were prepared and *TOP1* knockdown (KD) was verified by western blotting.

### 2.4. Protein Isolation and Western Blotting

To analyze protein levels from whole cells, total protein was extracted from cell samples using 1X Cell Lysis Buffer (Cell Signaling Technology, Danvers, MA, USA) supplemented with 1X Halt™ Protease and Phosphatase Inhibitor Cocktail (Thermo Fisher) and 1X PhosSTOP Phosphatase Inhibitor Cocktail (Roche, Indianapolis, IN, USA). Protein concentration of the extracts was assessed using the colorimetric Protein Assay Dye Reagent (Bio-Rad, Hercules, CA, USA) according to the manufacturer’s instructions, with absorbance at 590 nm detected by the SpectraMax M3 plate reader (Molecular Devices). Aliquots of 10 μg of protein per sample were denatured with 1X NuPAGE^®^ LDS Sample Buffer (Thermo Fisher) by heating at 95 °C for 5 min. Samples were loaded onto 4–20% gradient TGX gels (Bio-Rad), and proteins were separated by size using gel electrophoresis. Proteins were transferred to 0.2 μm nitrocellulose membranes (Bio-Rad) using the Trans-Blot Turbo system (Bio-Rad). The membranes were blocked in 5% non-fat milk powder dissolved in 0.1% Tween-20 in Tris-buffered saline (TBST). The membranes were incubated with primary antibody, then HRP-conjugated secondary antibody, both diluted in 1% non-fat milk powder dissolved in TBST. Chemiluminescent signal was developed using either SuperSignal™ West Dura substrate (Thermo Fisher) or Clarity Max™ Western ECL Substrate (Bio-Rad) and visualized on the ChemiDoc imaging system (Bio-Rad). The following primary antibodies were obtained from Cell Signaling Technology: GAPDH (#2118, diluted 1:5000), p-FAK (Tyr397; #8556, diluted 1:1000), FAK (#13009, diluted 1:1000, p-H2AX (Ser139; #2577, diluted 1:1000), and histone H3 (#5192, diluted 1:1000). TOP1 antibody was obtained from BD Biosciences (#556597, diluted 1:1000). HRP-conjugated secondary antibodies recognizing mouse (#7076, diluted 1:2000) and rabbit (#7074, diluted 1:2000) proteins were purchased from Cell Signaling Technology.

### 2.5. Cell Confluence Assays

Cells were seeded onto 384-well plates and treated 24 h later with either serial or incremental dilutions of a single drug. Cell confluence was measured by the Incucyte^®^ S3 Live-Cell Analysis System (Sartorius, Göttingen, Germany). Most assays were conducted for 3–5 days. Graphed data generally corresponds to a time point when the vehicle-treated cells reached an average confluence of 90–95%. Compounds tested in this assay were either purchased from Selleckchem (CPT and GNF-7) or generously gifted by Dr. Craig Thomas (National Center for Advancing Translational Sciences (NCATS), NIH; losmapimod, talmapimod and ralimetinib) or Dr. Marielle Yohe (NCI, NIH; SB203580).

### 2.6. Synergy Assay

Cells were seeded onto 384-well plates, allowed to attach for 24 h, then treated with a combination of various concentrations of CPT and GNF-7. Cell viability was measured after 5 days of treatment by an MTS assay (Promega). The cell viability data was used as the input into the SynergyFinder 1.0 R package, and this tool was used to calculate and generate Bliss synergy plots [23].

### 2.7. Cell Doubling Time

Equal numbers of EW8 and *TOP1* KD cells were plated in multiple wells of 12-well plates. Each day, two replicates of each cell line were trypsinized, stained with trypan blue and viable cells were counted using a Cellometer Auto 2000 (Nexcelom Bioscience, Lawrence, MA, USA). Doubling time was calculated during the linear growth phase (between days 3 and 4) using the formula: doubling time = T × ln(2)/ln(X_e_/X_b_), where T is the incubation time, and X_e_ and X_b_ are the number of cells at the end and the beginning of the incubation time, respectively.

### 2.8. Exome Sequencing

Genomic DNA from 1 × 10^6^ untreated cells was extracted with the QIAamp DNA Mini kit (Qiagen) according to the manufacturer’s protocol. Exome sequencing was performed by the Center for Cancer Research Sequencing Facility (NCI, NIH). Libraries were prepared using the SureSelect XT kit from Agilent, and the resulting libraries were sequenced on Illumina’s NovaSeq instrument using paired-end sequencing and a read length of 150 bp.

### 2.9. RNA Sequencing

EW8 and *TOP1* KD cells were either left untreated or treated with 40 nM GNF-7 and collected at various time points after treatment. Two biological replicates were collected for each time point from each cell line. Cell pellets were resuspended in Buffer RLT and homogenized by passage through QIAshredder columns (Qiagen). Total RNA was extracted with the Qiagen RNeasy Plus Mini kit by following the manufacturer’s instructions. The Center for Cancer Research Sequencing Facility performed RNA quality checks and RNA sequencing. RNA quality and quantity was assessed on an Agilent 2100 Bioanalyzer. Libraries were prepared using the TruSeq Stranded mRNA Library Prep kit (Illumina) and then sequenced on a HiSeq4000 sequencer (Illumina) with paired-end sequencing and a read length of 150 bp.

### 2.10. RNA-Seq and Exome-Seq Data Analysis

For RNA sequencing data, reads were trimmed for adapters and low quality bases using Trimmomatic 0.38 software. Data normalization and statistical analysis were performed using Partek Flow (St. Louis, MO, USA) on the NIH Helix cluster (https://partekflow.cit.nih.gov; accessed 5 June 2019). Sequencing reads were aligned to *hg19* genome using STAR-2.4.1d aligner, and transcript abundance was estimated by employing the Partek E/M algorithm based on RefSeq Transcripts 87. Gene specific reads were normalized to total read count per sample and differentially expressed genes were identified using the differential gene expression (GSA) algorithm. GSEA (https://www.gsea-msigdb.org/gsea/index.jsp; accessed 15 and 21 July 2019) [24] and DAVID functional annotation tools [25] were used for downstream analysis. For exome sequencing, read mapping and variant calling were done using the NCI Collaborative Bioinformatics Resources CCBRPipeliner (http://ccbr.github.io/Pipeliner; accessed 22 September 2019) available on the NIH’s Biowulf cluster.

### 2.11. Quantitative High-Throughput Combination Screening (qHTCS)

Single agent drug screening was performed as previously described by the Division of Preclinical Innovation (NCATS, NIH) [26,27]. Briefly, 10 nL of compounds were dispensed into 1536-well tissue culture-treated plates using an Echo 550 acoustic liquid handler (Labcyte, San Jose, CA, USA). Cells were added at 500 cells/well in 5 μL of medium. An 11-point custom concentration range was generated for all of the NCATS Mechanism Interrogation Plate (MIPE) 5.0 drugs (range: 56 mM to 0.7 nM). Plates were incubated for 48 h in standard incubator conditions. At 48 h post cell addition, 3 μL of CellTiter-Glo^®^ was added to all wells and plates were incubated for 15 min at room temperature. Luminescence readings were taken with a 2 s exposure time per plate using a Viewlux imager (PerkinElmer, Waltham, MA, USA).

### 2.12. Capillary Immunoassays

The Simple Western™ (ProteinSimple, San Jose, CA, USA) automated capillary electrophoresis immunoassay system was utilized. Cells were treated as indicated, pelleted, and flash frozen. Cell lysates were prepared in 1X Cell Lysis Buffer (Cell Signaling Technology) supplemented with 1X Halt™ Protease and Phosphatase Inhibitor Cocktail (Thermo Fisher) and 1X PhosSTOP Phosphatase Inhibitor Cocktail (Roche). Protein separation, immunodetection, and analysis steps were performed automatically as previously described [28]. Protein bands were analyzed with the Compass software 5.0.1 (Bio-Techne, Minneapolis, MN, USA) and normalized to the housekeeping gene GAPDH.

### 2.13. Cell Proliferation and Apoptosis Induction Assays

Cell proliferation and viability were measured using the CellTiter 96^®^ AQ_ueous_ One Solution Cell Proliferation Assay from Promega according to the manufacturer’s directions. Briefly, 20 μL of MTS reagent was added to cell sample wells containing 100 μL of media, then incubated at 37 °C for 2 h. Absorbance at 490 nm was measured using a SpectraMax M3 plate reader. Measurement of caspase-3 and caspase-7 activities was used as a surrogate measurement for the induction of apoptosis. Cells (1 × 10^4^) were plated in white, clear-bottom 96 well plates, allowed to attach for 24 h, then treated with various drugs or with DMSO vehicle for 24 h. Caspase-3 and caspase-7 activity was measured by utilizing the Caspase-Glo^®^ 3/7 Assay reagent (Promega), following the manufacturer’s protocol. After 30 min of incubation at room temperature (RT), luminescence was measured with the SpectraMax M3 plate reader.

### 2.14. Cell Cycle Analysis

The cell cycle distribution of EW8 and EW8 *TOP1* KD cells after treatment with GNF-7, CPT, or vehicle (DMSO) for 24, 48 or 72 h was analyzed by staining cells with propidium iodide (PI) and then measuring individual cell fluorescence by flow cytometry. Culture media and trypsinized, treated cells were collected, washed first with PBS, then with 1% FBS in PBS, and then fixed in ice cold 70% ethanol. For staining, cells were washed with 1% FBS in PBS and then resuspended in PBS. Phosphate-citric acid buffer (0.2 M Na_2_HPO_4_/0.1M citric acid pH 7.8) was added and the cell mixture was incubated at RT for 5 min. Cells were spun down and resuspended in 500 μL of a freshly prepared PI/RNase solution (0.2 mg/mL RNase and 20 μg/mL PI (both from Millipore Sigma) in 1% FBS in PBS), then incubated for 15 min at 37 °C. The fluorescent signal of the stained cells was analyzed by flow cytometry on an LSRFortessa (BD Biosciences). FlowJo software (BD Biosciences) was used to plot histograms of fluorescent intensity and to calculate the percentages of cells in each phase of the cell cycle with the Watson model.

### 2.15. Fluorescent Cell Cycle Reporter Assay

EW8 cells were infected with Incucyte^®^ Cell Cycle Green/Red Lentivirus (Sartorius), which contains a cassette that expresses GFP during the S/G2/M cell cycle phases and mKate2 (a red fluorescent protein) during G1. Successfully infected cells were selected with 0.25 μg/mL puromycin. These cells were seeded onto 96-well plates in four biological replicates and then treated with DMSO, CPT or GNF-7 24 h later. The Incucyte^®^ S3 Live-Cell Analysis System was used to obtain red and green fluorescent images every 30 min from the time of plating through 72 h of drug treatment. The Incucyte^®^ S3 software (Sartorius) was then used to calculate the percentage of red, green, and yellow cells out of the total cells detected for each time point.

### 2.16. In Situ Kinase Selectivity Profiling (KiNativ™)

EW8 and EW8 *TOP1* KD cells were treated with DMSO or 40 nM GNF-7 for 1 h or 24 h or with 400 nM GNF-7 for 1 h and cells were collected in PBS. Cells were lysed, and the lysates were labeled with probe and analyzed by LC/MS-MS using the KiNativ platform (ActivX, La Jolla, CA, USA) as previously described [29]. Mass spectrometry results from the GNF-7-treated lysates were compared to the DMSO lysates from the corresponding time point to determine the percent inhibition of each kinase. The online tool KinMap was used to plot the results onto a kinase tree [30].

### 2.17. siRNA Kinome Screen

The Silencer Select Human Kinase siRNA library (Thermo Fisher Scientific), which targets 709 human kinase genes, was used for primary screening. Screening was performed by the Functional Genomics Lab (NCATS, NIH). Briefly, three siRNAs per gene were arrayed in 384 well plates, one siRNA per well. For each well, 2 μL of siRNA was complexed with 0.07 μL RNAiMax Lipofectamine transfection reagent (Invitrogen, Carlsbad, CA, USA) in 20 μL of serum-free RPMI-1640 media for 30 min at room temperature. Two thousand five hundred EW8 and EW8 *TOP1* KD cells resuspended in 20 μL of RMPI-1640 and 20% fetal bovine serum (Millipore Sigma) was added. Cell viability was measured by using the CellTiter-Glo^®^ assay and a BMG Pherastar plate reader (Ortenberg, Germany) following the manufacturer’s instructions 72 h after siRNA transfection.

### 2.18. Immunofluorescence Staining and Microscopy

EW8 and EW8 *TOP1* KD cells were plated at low density (3 × 10^3^) onto 8-well polymer μ-slides (ibidi, Gräfelfing, Germany) and given 72 h to attach. The cells were then treated with either DMSO or 40 nM GNF-7. Cells were fixed with 4% methanol-free formaldehyde (Thermo Fisher), permeabilized with 0.1% Triton-X 100, and blocked with 3% bovine serum albumin (BSA). The cells were then stained with Alexa Fluor Plus 647 phalloidin (Thermo Fisher; diluted 1:400) and Alexa Fluor 488-conjugated vinculin antibody (Abcam (Cambridge, UK); ab196454; diluted 1:100) for 1 h at RT. The nuclei were counterstained with DAPI (Thermo Fisher) and VectaShield^®^ Plus Antifade Mounting Medium (Vector Laboratories, Newark, CA, USA) was added to all wells. Images were acquired under identical parameters at 60X using a Zeiss LSM 780 confocal microscope (Oberkochen, Germany). Phase microscopy images were taken using an Incucyte S3 (Sartorius) using a 10X objective.

### 2.19. Immunohistochemistry and H&E Staining

IHC and histology staining were performed by the Molecular Histopathology Laboratory (Frederick National Laboratory for Cancer Research). IHC staining was performed on Leica Biosystems’ BondRX autostainer with the following conditions: Isotype control reagent was used in place of the primary antibodies for the negative controls. Epitope Retrieval 1 (Citrate), 10 min, was used for biotin-conjugated p-histone H2AX (Ser139; Millipore #16–193, diluted 1:100, 60 min) and staining was completed using the Bond Intense R Detection Kit (Leica Biosystems, Nussloch, Germany). H&E and IHC sections were scanned at 20X using an Aperio AT2 scanner (Leica Biosystems) and analysed using the NCI HALO Image Analysis Resource. Tumor area was manually annotated by a veterinary pathologist. Image analysis was performed using CytoNuclear v2.0.5 algorithm in HALO v3.2 (Indica Labs, Albuquerque, NM, USA) to determine the percentage of positive p-H2AX cells.

### 2.20. Animal Models

All animal experiments were approved by and performed in accordance with the guidelines of the National Institutes of Health Animal Care and Use Committee (Bethesda, MD, USA). Approximately 2 × 10^6^ EW8 cells suspended in a 1:1 solution of Matrigel (Corning, Corning, NY, USA) and Hank’s Balanced Salt Solution (VWR, Radnor, PA, USA) were injected into the left hind leg gastrocnemius muscle of 6-week-old female Fox Chase SCID-Beige mice (CB17.B6-Prkdcscid Lystbg/Crl) (Charles Rivers Laboratories, Wilmington, MA, USA). As soon as tumors were palpable, mice were randomized into four groups of 11. Group A was treated with IP and oral gavage vehicles, group B was treated with 1.25 mg/kg irinotecan and oral gavage vehicle, group C was treated with 10 mg/kg GNF-7 and IP vehicle, and group D was treated with 1.25 mg/kg irinotecan and 10 mg/kg GNF-7. Irinotecan (Pharmacia & Upjohn Co, Peapack, NJ, USA) was diluted in sterile saline (Fresenius Kabi, Bad Homburg, Germany) and administered by intraperitoneal injection, while GNF-7 (Tocris, Bristol, UK) was suspended in a solution of 5% 1-Methyl-2-pyrrolidinone (NMP)/15% solutol/30% PEG400/50% 0.05 M citric acid (all from Millipore Sigma) and administered by oral gavage. Treatment began the same day that mice were randomized. Irinotecan was given once daily on days 1–8, and GNF-7 was given once daily on days 6–8. Tumor size was measured twice weekly with calipers, starting on Day 1 of treatment and continuing until tumors reached study endpoint size. The health of the mice was monitored throughout by observation and weekly weighing. Tumor volume was calculated using the formula: V = (D × d^2^)/6 × 3.14, where D is the longer tumor axis and d is the shorter tumor axis. Tumors were harvested from 3 mice per group on Day 8 of treatment and from the remainder of the mice when the tumors reached endpoint size.

### 2.21. Statistical Analysis

Dose response curves were fitted using least squares regression with four parameters. Simple western and casp3/7 activation assay statistics were done using two-way ANOVA with Dunnett’s multiple comparisons test (to compare treated to untreated values within the same cell type) or Šídák’s multiple comparison test (to compare EW8 to TOP1 KD at each time point). Analysis of IHC and histology quantification was performed with one-way ANOVA with Tukey’s multiple comparison’s test. Kaplan–Meier survival curves were compared using the Gehan–Breslow–Wilcoxon test. Data were analyzed and plotted using GraphPad Prism 9 software (Boston, MA, USA) unless otherwise noted in the methods.

## 3. Results

### 3.1. TOP1 Knockdown Confers Resistance to TOP1 Inhibitors in ES Cells

**Figure 1:**
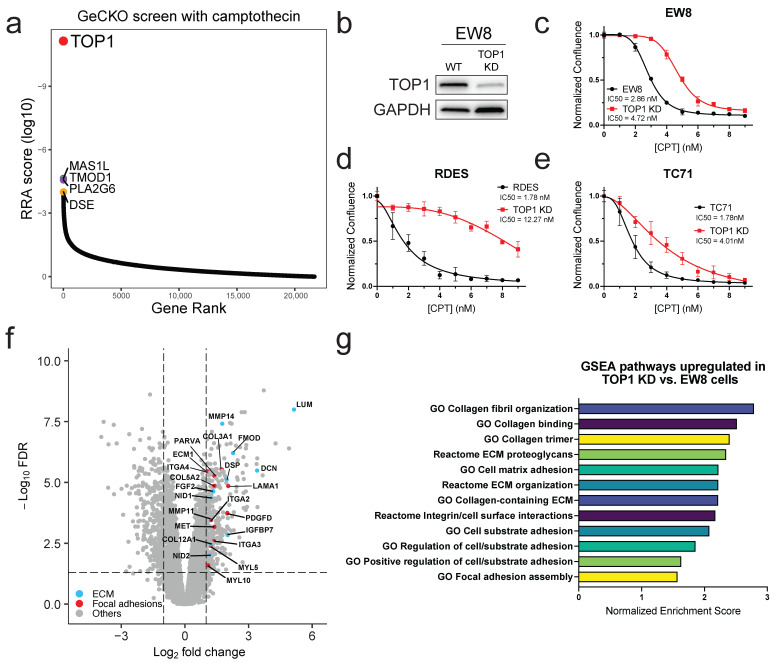
A genome-wide CRISPR screen reveals *TOP1* deletion as a primary resistance mechanism to camptothecin. (**a**) Distribution of RRA scores of sgRNAs enriched in EW8 cells treated with CPT (8 nM) vs. DMSO from a genome-wide CRISPR library screen (*n* = 4 biological replicates); (**b**) Western blot of TOP1 expression in EW8 and EW8 *TOP1* KD cells; (**c**–**e**) dose response curves for EW8 (**c**), RDES (**d**), and TC71 (**e**) cells and their corresponding *TOP1* KD cells treated with various doses of CPT for 3.25 d (EW8 and RDES) or 4.25 d (TC71). Data shown are mean ± SD (*n* = 3–4 replicates). Graphs shown are representative data from one of three to four independent experiments; (**f**) Volcano plot showing differentially expressed genes in *TOP1* KD cells vs. parental EW8 cells (*n* = 2–3 biological replicates). Blue and red dots represent genes associated with the extracellular matrix and focal adhesions, respectively (DAVID enrichment analysis). Dashed lines at FDR = 0.05 and fold change = ±2; (**g**) bar graph of normalized enrichment scores (NES) of gene sets related to cell adhesion that are positively enriched in *TOP1* KD cells vs. EW8 cells by GSEA analysis. All scores are considered significant (FDR q-value < 0.25).

To identify genes whose knockout grants resistance to topoisomerase I (TOP1) inhibitors, we employed a positive selection genome-wide CRISPR-Cas9 knockout screen using the GeCKO v2 sgRNA library [21]. EW8 ES cells were infected at a low multiplicity of infection (MOI) of ~0.3, and successfully transduced cells were selected with puromycin for 10–11 days. Following selection, cells were divided into two groups and one group was treated with a dose of the TOP1 inhibitor camptothecin (CPT) that had previously been determined to be sufficient to kill all EW8 (8 nM), while the other was treated with DMSO as a vehicle control. Cells from both groups were collected after 27 days of treatment. The abundance of sgRNAs in each treatment group was determined by next generation sequencing. The MAGeCK algorithm was used to compare the CPT-treated cells to the DMSO-treated cells to determine which sgRNAs had been augmented in the CPT-treated cells [22]. When the results from the four biological replicates were combined, only *TOP1* was identified as being significantly positively enriched in EW8 cells after treatment with CPT (Figure 1a).

To verify that *TOP1* knockout induced resistance to TOP1 inhibitors, we used a CRISPR-Cas9 lentiviral vector that contained an sgRNA targeting TOP1 to knockout *TOP1* in EW8 cells. We isolated individual clones and then identified those that were successfully edited using immunoblotting (Figure 1b). Interestingly, we found that we were unable to completely ablate all TOP1 protein expression in any of the clones tested. Therefore, the clone that we chose to perform all of the following experiments will be referred to as *TOP1* KD (knockdown). We further verified KD of *TOP1* in this clone by performing exome sequencing on the WT and *TOP1* KD EW8 cells and found that there was a 20 bp deletion in exon 3 of the *TOP1* gene in the *TOP1* KD cells that resulted in a frame shift and a premature stop codon (Supplementary Figure S1a). Once we had the *TOP1* KD cells generated and validated, we confirmed that depletion of *TOP1* did in fact confer resistance to TOP1 inhibitors by monitoring the growth of the *TOP1* WT and the *TOP1* KD EW8 cells after treatment with CPT and plotting the confluence to generate dose response curves. Indeed, loss of *TOP1* resulted in a shift to a higher IC50 for CPT compared to the *TOP1* WT EW8 cells (Figure 1c). We performed cell counting experiments and found that the doubling time in the *TOP1* KD cells was comparable to the WT, indicating that knocking down *TOP1* did not cause any substantial growth defects (Supplementary Figure S1b). In addition, no differences were seen in γ-H2AX levels between the EW8 and *TOP1* KD cells when treated with CPT for 24 h (Supplementary Figure S1c). The same effect on cell growth was seen when these two cell lines were treated with another TOP1 inhibitor, topotecan (Supplementary Figure S1d). We then demonstrated that this effect was not unique to EW8 cells by knocking down *TOP1* in two additional ES cell lines, RDES and TC71, and performing dose response experiments (Figure 1d,e and Supplementary Figure S1e). Again, knocking down *TOP1* increased the IC50 for CPT in both lines when compared to the *TOP1* WT parental cells.

To further characterize the differences between EW8 WT and TOP1 KD cells, we performed RNA sequencing of untreated cells from each cell line and identified the differentially expressed genes. A total of 309 genes were significantly upregulated (FDR ≤ 0.05) by two-fold or more in the *TOP1* KD cells compared to the EW8 WT cells, while 339 genes showed a significant decrease in expression by at least two-fold. Analysis of the genes significantly upregulated by two-fold or more in the *TOP1* KD cells with the functional annotation tool DAVID demonstrated an enrichment of genes associated with Rho GTPase signaling, collagen fibril organization and extracellular matrix, and focal adhesions (Figure 1f). Similarly, when we analyzed all significantly differentially expressed genes (both up- and downregulated) in the *TOP1* KD cells with Gene Set Enrichment Analysis (GSEA), terms such as GO extracellular matrix component, GO collagen binding and Reactome signaling by Rho GTPases were identified as being positively enriched in the *TOP1* KD cells (Figure 1g and Supplementary Figure S1f) [24,31].

### 3.2. Small-Molecule Screening Identifies GNF-7 as Having Increased Potency in TOP1-Deficient Cells

**Figure 2:**
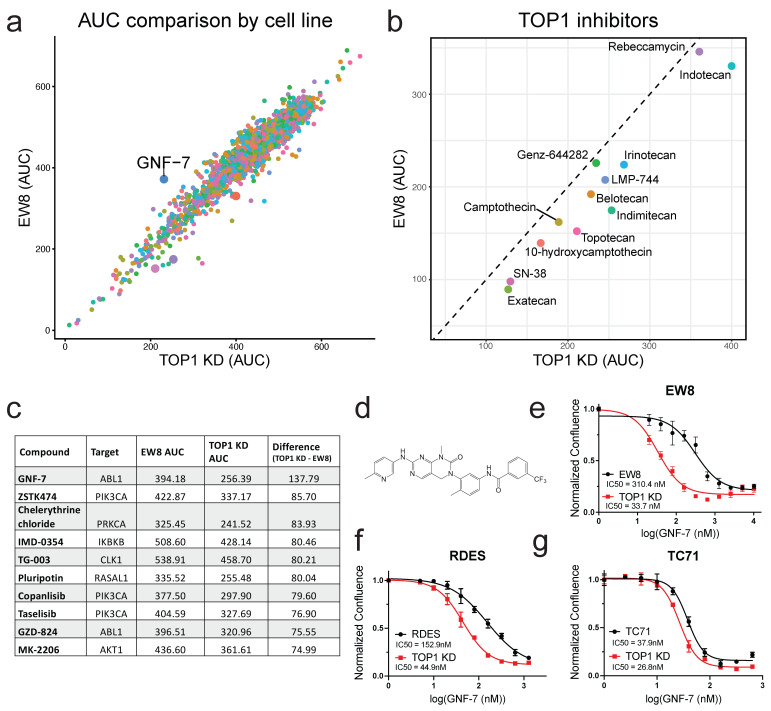
GNF-7 is preferentially cytotoxic to EW8 *TOP1* KD cells compared to parental cells. (**a**) Scatterplot of areas under the curve (AUC) of dose response curves for EW8 vs. EW8 *TOP1* KD cells treated with compounds in the MIPE small-molecule library (*n* = 2480 compounds); (**b**) scatterplot highlighting TOP1 inhibitors from the data shown in (**a**). Dashed line at y = x. (**c**) Table of the AUC values for each cell line for the ten compounds with the greatest difference in AUC for *TOP1* KD cells compared to EW8 cells. Also included are the putative targets of these compounds. (**d**) Chemical structure of GNF-7 (upper left). (**e**–**g**) Dose response curves for EW8, RDES, and TC71 cells and their corresponding *TOP1* KD cells treated with various doses of GNF-7 (upper right, lower left, and lower right, respectively) for 4.5 d (EW8 and RDES) or 4.25 d (TC71). Data shown as mean ± SD (*n* = 3–5 replicates; representative data from one of three to five independent experiments).

Once we had established that the EW8 *TOP1* KD cells were an effective model for TOP1 inhibitor resistance, we sought to identify compounds with enhanced cytotoxic effects in the *TOP1* KD cells compared to the parental EW8 cells. Utilizing a high throughput chemical library screen, we tested the viability of the EW8 and *TOP1* KD cells when treated with various doses of drugs from the MIPE library, a small-molecule library containing clinically relevant compounds. By comparing the area under the curve (AUC) for the dose response curves generated for each compound in each cell line, we identified drugs that had a greater effect on viability in the *TOP1* KD cells, those with a greater effect in the EW8 WT cells, and those that did not exhibit a differential effect between the two lines (Figure 2a). The majority of the compounds tested demonstrated little difference in effectiveness between the two cell lines. As anticipated, nearly all of the TOP1 inhibitors included in the library showed more cytotoxic activity in the parental EW8 cells (Figure 2b). We also found that, of the drugs that showed increased cytotoxicity in the *TOP1* KD cells, there was an enrichment of AKT1 and PI3KCA inhibitors (Supplementary Figure S2a).

Despite an abundance of AKT1 and PI3KCA inhibitors in the list of drugs that had higher toxicity in the *TOP1* KD cells, the drug with the largest differential effect, GNF-7, was originally developed as a BCR-ABL kinase inhibitor (Figure 2c,d; [18]). However, we found that ABL1 inhibition was unlikely to solely responsible for the enhanced sensitivity of the *TOP1* KD cells to GNF-7, since further examination of other ABL1 inhibitors included in the screen showed that there was no difference in effect of these drugs between the two cell lines (Supplementary Figure S2b). In fact, alternative ABL1 inhibitors had little to no effect on cell viability of either cell line, having IC50 values of at least ten-fold greater than the IC50 value of GNF-7 in the parental EW8 cells. Additionally, we independently confirmed this finding in cell growth assays to determine the dose response of the EW8 WT and *TOP1* KD cell lines to dasatinib, another BCR-ABL inhibitor, and found that there was no difference in cytotoxicity between the two (Supplementary Figure S2c).

We then validated that the *TOP1* KD cells did have increased sensitivity to GNF-7 compared to the WT cells by treating the two cell lines with serial dilutions of GNF-7 and utilizing in vitro cell growth assays to determine the dose response of each cell line, and indeed we found that the IC50 for GNF-7 in EW8 parental cells was almost an order of magnitude higher than in the *TOP1* KD cells (310 nM vs. 34 nM, respectively; Figure 2e). We also showed that knocking down *TOP1* increases sensitivity to GNF-7 in both RDES and TC71 cells (Figure 2f,g). However, when we tested to see if the combination of GNF-7 and CPT is synergistic, Bliss score plots revealed that while there was synergistic activity at low doses of CPT in EW8, there was minimal synergy in the RDES and TC71 cells (Supplementary Figure S2d).

### 3.3. GNF-7 Increases G1 Arrest and Inhibits the EWS/FLI1 Gene Signature

**Figure 3:**
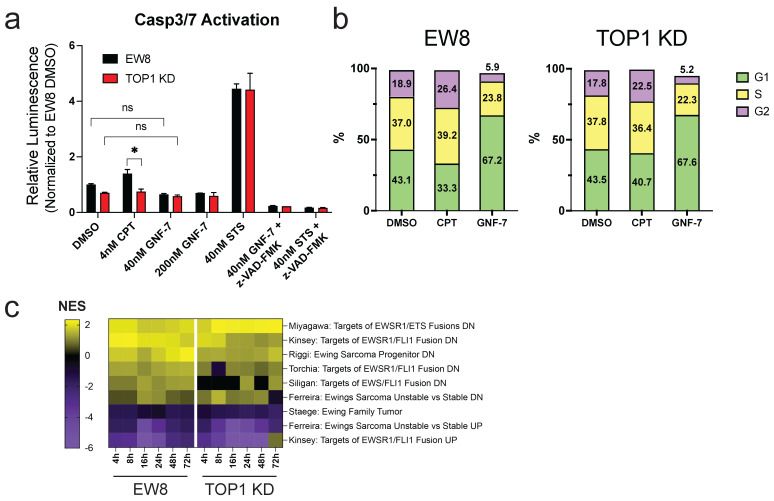
GNF-7 induces G1/S cell cycle arrest and reverses ES-associated gene signatures. (**a**) Levels of caspase-3 and -7 activity in EW8 and *TOP1* KD cells after 24 h of treatment with the indicated compounds. Error bars represent SD of two biological replicates. *, *p* < 0.05; ns, not significant (two-way ANOVA). (**b**) Cell cycle distribution of EW8 (left) and *TOP1* KD (right) cells treated with either DMSO, 4 nM CPT, or 40 nM GNF-7 for 24 h. Numbers within the bars represent the percentage of cells in that phase. (**c**) Heat map of GSEA NES for gene sets related to ES in EW8 and *TOP1* KD cells based on RNA expression after treatment with 40 nM GNF-7 for various times. For (**a**,**b**), data shown are representative data from one of two independent experiments.

Having identified GNF-7 as a small molecule that has higher toxicity in *TOP1* KD cells, our model system for TOP1 inhibitor resistance, we performed several experiments to illustrate its effects on EW8 *TOP1* WT and *TOP1* KD cells. We chose to perform most experiments with 40 nM GNF-7, a dose close to the IC50 of the *TOP1* KD cells where differential viability of the two cell lines is readily evident. First, we established that GNF-7 did not stimulate apoptosis in EW8 cells, regardless of *TOP1* status. Treating either cell type with a low (40 nM) or high (200 nM) dose of GNF-7 did not induce caspase3/7 activity above the baseline of vehicle-treated cells (Figure 3a). Next, we examined whether GNF-7 induced cell cycle arrest using two different approaches. One method we employed was to stain cell nuclei with propidium iodide followed by flow cytometry analysis to quantify the amount of DNA in each cell. We found that in both EW8 WT and *TOP1* KD cells treated with GNF-7 for 24, 48 or 72 h, GNF-7 caused a substantial accumulation of cells in the G1 phase of the cell cycle (Figure 3b and Supplementary Figure S3a,b). We also saw induction of G2/M arrest in both cell lines when treated with CPT, which agrees with previously published data (Figure 3b and Supplementary Figure S3a,b). We then confirmed these observations using EW8 cells expressing a Fucci cell cycle reporter system and again found that GNF-7 potently induced G1/S arrest (Supplementary Figure S3c).

We also examined the transcriptomic effects of low dose GNF-7 treatment on both EW8 parental and *TOP1* KD cells. We extracted RNA from EW8 WT and *TOP1* KD cells treated with GNF-7 for 0, 4, 8, 16, 24, 48 or 72 h and performed mRNA sequencing. Interestingly, we found that treatment with GNF-7 promoted the expression of genes that are normally downregulated by the expression of the EWS::FLI1 oncogene, while decreasing expression of genes typically induced by EWS::FLI1 (Figure 3c, Supplementary Figure S3d). In addition, in both *TOP1* WT and *TOP1* KD cells, GNF-7 treatment decreased the expression of genes associated with the genes upregulated by EWS::FLI1 (Figure 3c).

### 3.4. GNF-7 Inhibits Multiple Kinases

**Figure 4:**
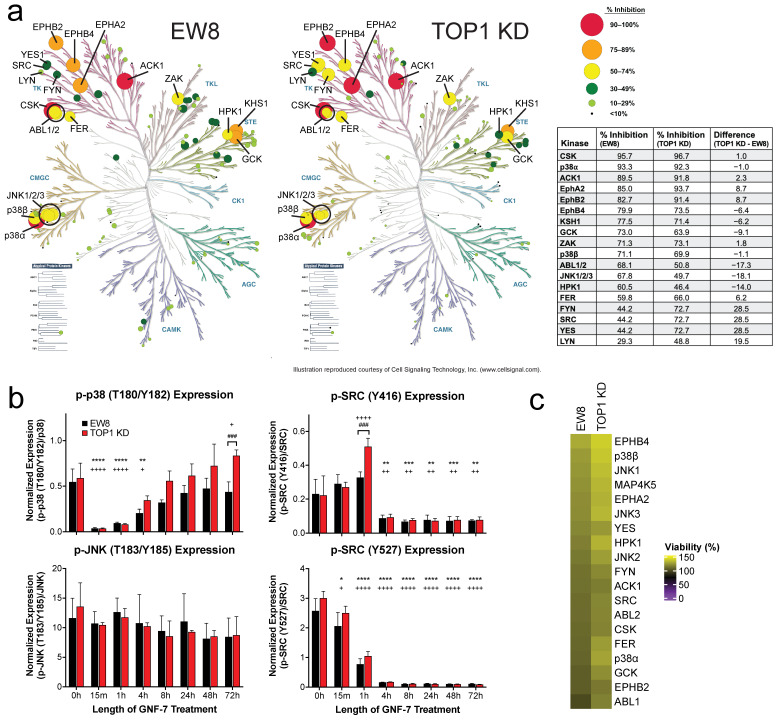
GNF-7 potently inhibits p38α and several tyrosine kinases. (**a**) Kinase trees mapping in situ kinase inhibition data from EW8 (left) and *TOP1* KD (center) cells treated with 40 nM GNF-7 for 1 h. Circle color and size indicate percent inhibition. Table (right) displays all kinases inhibited >50% in either cell line, with the actual percent inhibition values for both cell lines, as well as the difference in inhibition between the two lines. Kinase tree illustrations reproduced courtesy of Cell Signaling Technology, Inc. (**b**) Protein expression of phosphorylated p38α (upper left), JNK (lower left), and SRC (upper and lower right) in EW8 and *TOP1* KD cells after treatment with 40 nM GNF-7 for various lengths of time. All data normalized to the corresponding unphosphorylated protein and shown as mean ± SD (*n* = 3). * or ^+^, *p* < 0.05; ** or ^++^, *p* < 0.01; *** or ^+++^, *p* < 0.001; **** or ^++++^, *p* < 0.0001 (value compared to 0 h for EW8 (*) or *TOP1* KD (^+^) by two-way ANOVA with Dunnett’s multiple comparisons test). ^###^, *p* < 0.001 (*TOP1* KD value compared to EW8 at that time point by two-way ANOVA with Šídák’s multiple comparison test). (**c**) Heat map of the percentage of viable EW8 or *TOP1* KD cells 72 h after transfection with siRNAs targeting the kinases identified in (**a**). Each data point represents the average value of three individual siRNAs.

To further elucidate the mechanism of action of GNF-7 in our cell lines, we utilized in situ kinome profiling to identify the kinases bound by GNF-7 in both EW8 and *TOP1* KD cells. We examined kinase binding by GNF-7 after short and prolonged treatment times (1 h and 24 h) when cells were treated with a low dose of GNF-7 (40 nM), and 1 h with a high dose (400 nM). Twelve kinases were found to be inhibited by GNF-7 in both cell lines at all doses and time points, including CSK (C-terminal Src kinase), p38α (*MAPK14*), ACK1 (activated Cdc42-associated kinase; *TNK2*), GCK (germinal center kinase; *MAP4K2*), several ephrin receptors, and ABL1/2 (ABL proto-oncogene 1/2, non-receptor tyrosine kinase) (Figure 4a, Supplementary Figure S4a,b). After 1 h of treatment with 40 nM GNF-7, *TOP1* WT and KD cells exhibited similar kinase inhibition profiles, with one notable exception being that SRC (SRC proto-oncogene, non-receptor tyrosine kinase), Fyn (FYN proto-oncogene, Src family tyrosine kinase), and YES1 (YES proto-oncogene 1, Src family tyrosine kinase) had higher inhibition in the *TOP1* KD cells (Figure 4a). Several additional kinases were inhibited in both cell lines when treatment was extended to 24 h, such as Lyn (LYN proto-oncogene, Src family tyrosine kinase), ZAK (zipper sterile-alpha-motif kinase; *MAP3K20*) and TAO2 (TAO kinase 2; *TAOK2*), as well as some cell cycle-dependent kinases like Aurora B (*AURKB*) and PLK1 (polo like kinase 1), all of which we attributed to secondary effects of the kinases initially inhibited by GNF-7 (Supplementary Figure S4a). We then used the Simple Western system to validate the KiNativ results by examining the phosphorylation/activation status of several of the discovered GNF-7 targets. When EW8 WT and *TOP1* KD cells were treated with GNF-7 for various times, an immediate (15 min) decrease in p38α phosphorylation at Thr180/Tyr182 was induced, which then gradually increased back to baseline levels by 24 h (Figure 4b). While no significant changes were seen in phosphorylated JNK1/2/3 (c-Jun N-terminal kinase 1/2/3; *MAPK8/9/10*) (Thr183/Tyr185), there was a significant downregulation of phosphorylated SRC at two different sites. At Tyr416, GNF-7 promoted a sustained inhibition of phosphorylation starting at 4 h after treatment. Phosphorylation of SRC at Tyr527 decreased after 1 h of treatment, reaching a nadir at 4 h.

Finally, to discern which of the identified kinases was responsible for the cytotoxic effects of GNF-7, we executed a knockdown screen on our two cell lines using a human kinase siRNA library. While knockdown of several mitosis-related kinases induced a substantial reduction in cell viability in both lines, we were unable to identify any specific kinases whose knockdown induced increased cytotoxicity in the *TOP1* KD cells compared to the EW8 parental cells (Supplementary Figure S4c). In fact, when we looked specifically at the kinases inhibited by more than 50% in at least one cell line after 1 h of GNF-7 treatment from the KiNativ assay, we not only failed to detect a differential cytotoxic effect between the cell lines, but we also found that knocking down these kinases had no effect on cell viability (Figure 4c).

### 3.5. GNF-7 Induces FAK Activation and Focal Adhesion Formation

**Figure 5:**
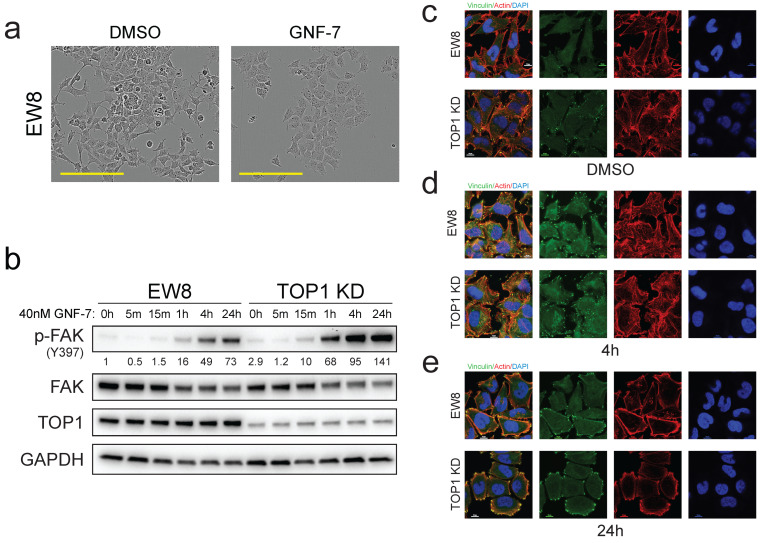
GNF-7 induces focal adhesions. (**a**) Phase microscopy images of EW8 cells treated with DMSO or 40 nM GNF-7 for 72 h. Scale bar, 200 μm. (**b**) Western blot showing FAK phosphorylation at Tyr397 after various lengths of treatment with 40 nM GNF-7. Numbers indicate densitometry values for p-FAK/FAK/GAPDH normalized to EW8 0 h. (**c**–**e**) IF staining of actin (red) and vinculin (green) in EW8 and *TOP1* KD cells treated with either DMSO for 48 h (**c**), or with 40 nM GNF-7 for 4 h (**d**) or 24 h (**e**). Scale bar, 10 μm.

While performing our in vitro experiments, we observed that GNF-7 induced distinct morphology changes that became more pronounced as the concentration increased (Figure 5a). To investigate this further, we blotted lysates from EW8 and *TOP1* KD cells treated with GNF-7 for various lengths of time for levels of phosphorylated focal adhesion kinase (FAK; *PTK2*). We observed that GNF-7 stimulated FAK phosphorylation at its auto-activation site (Tyr 397) by 1 h after treatment (Figure 5b). Intriguingly, this induction of phosphorylation by GNF-7 was higher in the *TOP1* KD cells compared to the *TOP1* WT cells. To determine whether this observation correlated to a functional change in the cells, we examined cell morphology and focal adhesions by immunofluorescent staining of actin and vinculin, respectively. In untreated cells, the actin cytoskeleton had a similar appearance in the EW8 WT and *TOP1* KD cells; however, *TOP1* KD cells had noticeably more focal adhesions than the parental EW8 cells (Figure 5c). Upon treatment with GNF-7 for 4 h, the presence of focal adhesions was significantly increased in both cell types (Figure 5d). In addition, we observed an induction of actin stress fibers in the EW8 cells, regardless of *TOP1* status (Figure 5d). After 24 h of GNF-7 treatment, the number of focal adhesions remained higher than at baseline in both cell lines, while the actin stress fibers were still present but had relocated to the periphery of the cells (Figure 5e).

### 3.6. Combination Treatment of GNF-7 and a TOP1 Inhibitor Extends Survival In Vivo

**Figure 6:**
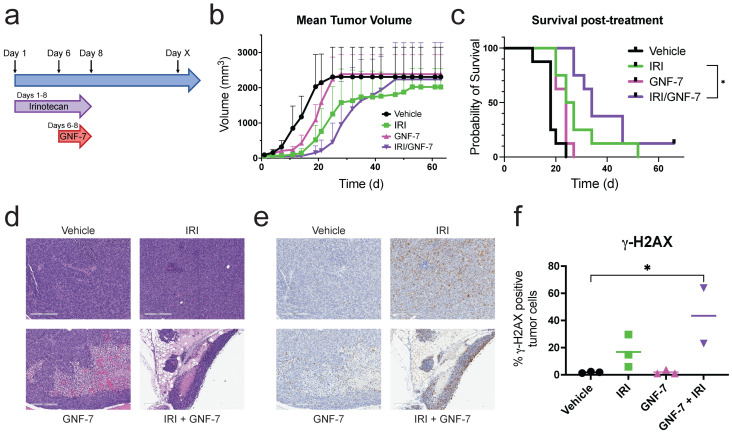
Combining GNF-7 and a TOP1 inhibitor improves survival in an orthotopic mouse model. (**a**) Diagram of the treatment schedule used for the in vivo experiment. (**b**,**c**) Average tumor volumes (**b**) and Kaplan–Meier survival curves (**c**) of mice orthotopically implanted with EW8 cells. Mice were treated with vehicle, irinotecan (1.25 mg/kg), GNF-7 (10 mg/kg) or the combination (*n* = 8–11 per treatment group). *, *p* = 0.0388 (Gehan–Breslow–Wilcoxon test). Data shown as average ± SD (**b**). (**d**) Representative histology images of EW8 tumors harvested 8 d after treatment initiation. (**e**) Representative IHC images for γ-H2AX staining from tumors harvested on day 8 of treatment. (**f**) Quantification of the γ-H2AX IHC data shown in (e) (*n* = 2–3). *, *p* < 0.05 (one-way ANOVA with Tukey’s multiple comparison test).

To ascertain whether combining GNF-7 with the TOP1 inhibitor irinotecan would provide a clinically relevant benefit in vivo, we utilized an ES model where EW8 cells were injected directly into the gastrocnemius muscle of mice. Mice were treated with either a low dose of irinotecan alone (1.25 mg/kg), GNF-7 alone (10 mg/kg), a combination of the two drugs, or vehicle only. Mice treated with irinotecan were treated for 8 consecutive days, while mice receiving GNF-7 were dosed on days 6–8 of the irinotecan treatment for a total of 3 treatments (Figure 6a). Both irinotecan and GNF-7 alone delayed the onset of exponential tumor growth compared to vehicle treated mice, while the combination of drugs delayed tumor growth even further (Figure 6b). We also found that the combination of irinotecan and GNF-7 significantly increased survival (*p* = 0.038) when compared to irinotecan alone, demonstrating the benefit of adding GNF-7 to the treatment regimen (Figure 6c).

To assess the effect of the treatments used on DNA damage signaling markers, we obtained tumor tissue from mice after treatment on the eighth day. H&E images revealed the small round blue cells characteristic of ES (Figure 6d). We also performed immunohistochemistry for γ-H2AX to detect the presence of DNA damage signaling, since TOP1 inhibitors like irinotecan are known to induce DNA damage. Interestingly, we discovered that while irinotecan alone increased γ-H2AX staining, albeit not significantly, the combination of GNF-7 and irinotecan resulted in a higher percentage of γ-H2AX positive cells. (Figure 6e,f).

## 4. Discussion

Understanding and targeting the molecular mechanisms of refractory disease after therapy remains among the most important challenges in pediatric oncology. Detailed understanding of these mechanisms could enable rational selection of therapeutic combinations that would anticipate and block pathways of tumor cell escape. In this work, we employed unbiased genetic and small-molecule screening to identify a multi-kinase inhibitor, GNF-7, that had unique potency in *TOP1*-deficient ES cells. Based on comprehensive kinome and molecular profiling, we discovered that GNF-7 exerts its effects by inhibiting or modulating multiple kinases including those that regulate focal adhesions. Finally, we confirmed that GNF-7 is effective in combination with low dose irinotecan in an ES orthotopic tumor mouse model, with significant extension of survival. While exciting, our findings suggest that further optimization of the formulation, schedule, and toxicity profiles of GNF-7 will be needed for translation of these findings into the clinic.

Clinically, TOP1i are frequently employed in relapse regimens of ES [3]. While single agent TOP1i’s have moderate single agent activity, synergy of these drugs with alkylating agents is a common strategy used to potentiate the clinical effect [32]. Unfortunately, despite responses, these relapse regimens are not generally curative and challenged by significant toxicity, most notably myelosuppression. The potential mechanisms of resistance to TOP1i have been extensively studied and can include point mutation of *TOP1* [33], downregulation of *TOP1* expression and stability [34], and upregulation of drug efflux pumps such as the ATP-binding cassette transporters [35]. The results of our genome-wide genetic screen suggest a strong selection for cells with genetic loss of *TOP1*. We do, however, acknowledge that due to the positive selection strategy used in our screen, we were unlikely to uncover many of the other potential TOP1i resistance mechanisms. Interestingly, ES cells appeared to tolerate the significant loss of TOP1 and demonstrated minimal change in their growth profiles even with a deficit of the protein. The large scale CRISPR screening efforts of DepMap (https://depmap.org/portal) identifies *TOP1* as a common essential gene and sheds light on why we were unable to generate any clones that had complete loss of TOP1 protein [36]. While cell growth did not appear to be limited in cells that had lost *TOP1*, we did note upregulation of several pathways associated with focal adhesions and matrix organization. While our conclusions are limited by the nature of the in vitro models used, it is notable that ES cell apoptosis and metastasis is known to be linked to the cytoskeletal reorganization orchestrated by EWS::FLI1 driven expression of ezrin and subsequent phosphorylation of focal adhesion kinase (FAK) [37,38].

While no kinases have been identified as mutated in ES, multiple kinases have been implicated for their oncogenic role including: IGF1R, VEGFR, EGFR, KIT and PDGFR [39,40]. Accordingly, multiple TKIs have been clinically studied, including in combination with chemotherapy, and demonstrating modest activity in selected patients with relapsed ES [41,42]. GNF-7 was originally identified as a Type II kinase inhibitor for BCR-ABL that delayed tumor growth in mouse model of chronic myelogenous leukemia [43,44]. Aside from its growth inhibition of BCR-ABL translocation-positive leukemias, GNF-7 has been shown to have a variety of effects in several other model systems [39,45,46]. One study demonstrated that GNF-7 inhibited the kinases ACK1 (activated Cdc42-associated kinase; *TNK2*) and GCK (germinal center kinase; *MAP4K2*) in an N-Ras mutant leukemia model, while another established that necroptosis in an acute kidney injury model could be prevented by GNF-7 via binding to RIPK1/3 (receptor interacting serine/threonine kinase 1/3) [39,40]. More recently, GNF-7 and its derivative SIJ1227 were shown to strongly decrease the proliferation, migration, and invasion of BRAF-mutant melanoma cells [41,42]. Our results using this compound in TOP1i resistant ES cells and their isogenic parental cells found that GNF-7 induces G1/S cell cycle arrest and reverses the transcriptional changes associated with EWS::FLI1 expression. Furthermore, we ascertained that a low dose of GNF-7 potently inhibits multiple kinases, including p38α (*MAPK14*), several ephrin receptors, ACK1, and CSK. We acknowledge that it is possible that the GNF-7 target in our system is outside to the profiled kinome and could potentially include non-kinase targets. It is intriguing, however, that our GNF-7 findings might align with previous work that discovered that focal adhesion kinase (FAK) is highly phosphorylated in Ewing sarcoma and small-molecule inhibition of FAK slows the growth of ES PDX models [47]. Indeed, we observed hyperphosphorylation of FAK with GNF-7 treatment and this effect was more pronounced in *TOP1*-deficient cells. However, our matching genetic knockout data suggests that GNF-7 likely induces its cytotoxic synergistic effects through a broader inhibition of multiple kinases, rather than by specifically targeting FAK. Given that inhibition of the YAP/TAZ/TEAD complex formation using verteporfin reduced stress fiber and focal adhesion formation and reduced metastatic ES [48], future studies will also focus on a potential role of GNF-7 in the ES metastatic process.

Our coupling of comprehensive genetic screening and isogenic cell lines with high-throughput small-molecule screening was effective in discovering GNF-7 and highlights that the strategy could be a model for the discovery of synthetically lethal small molecules. Conventionally, synthetic lethality refers to a combination of genes that when perturbed is lethal to the cell. Here, we explored the idea of synthetic lethality as a combination of a gene perturbation (*TOP1*) driven by a standard treatment, with a small molecule (GNF-7). The concept relies on the assumption that we can reliably predict the compensatory escape mechanism of the cancer cell but also leverages the ability of many pharmaceuticals, including tyrosine kinase inhibitors, to affect multiple targets. Our results suggest that the synthetically lethal chemical partner may be required to hit multiple targets rather than a single kinase or pathway to exert its maximal effect. Taken together, our study adds an exciting new direction to the current therapeutic arsenal for treatment of refractory pediatric tumors.

## 5. Conclusions

This study investigated the topoisomerase 1 inhibitor resistance in Ewing sarcoma cells. *TOP1*-deficient cells were notable for the upregulation of pathways involving focal adhesions and the extracellular matrix. Using unbiased small-molecule screening, the work discovered a small-molecule tyrosine kinase inhibitor, GNF-7, that had increased potency in *TOP1*-deficient cells. The kinase profiling of GNF-7 noted the inhibition of multiple kinases, including p38α (*MAPK14*), several ephrin receptors, ACK1, and CSK. These findings highlight a drug discovery strategy that could be a model for the discovery of synthetically lethal small molecules in refractory pediatric cancer.

## Data Availability

Sequencing data is deposited on the Gene Omnibus Expression (GEO) database, accession number: GSE298880.

## Supplementary Materials

The following supporting information can be downloaded at: https://www.mdpi.com/article/10.3390/cancers17152475/s1, Figure S1: TOP1 KD sensitizes ES cells to TOP1 inhibitors. (a) IGV figure showing exome sequencing reads over exon 3 of the TOP1 gene in parental EW8 and EW8 TOP1 KD cells; (b) Cell proliferation curves of untreated EW8 and TOP1 KD cells over 6 d (n = 2). Doubling time was calculated between days 3 and 4; (c) Western blot of *γ*-H2AX signal in EW8 and EW8 TOP1 KD cells treated with various doses of CPT for 24 h; (d) Dose response curves for EW8 and TOP1 KD cells treated with various doses of topotecan for 3 d (n = 4 replicates; error bars represent SD); (e) Western blots of TOP1 expression in RDES and RDES TOP1 KD cells (top) or in TC71 and TC71 KD cells (bottom). Representative data from one of two independent experiments (b-e); (f) GSEA plots of enriched gene sets in untreated TOP1 KD vs. EW8 cells. Plots correspond to the gene sets shown in Figure 1g; Figure S2: Pharmacological characterization of EW8 and TOP1 KD cells. (a) Scatterplot of area under the dose response curves (AUC) for EW8 and TOP1 KD cells, highlighting AKT and PIK3CA inhibitors. The GNF-7 data point is shown for reference. Dashed line at y = x; (b) Dose response curves for EW8 and TOP1 KD cells from the MIPE compound library screen for select drugs that target ABL1; (c) Dose response curves for EW8 and TOP1 KD cells treated with various doses of dasatinib for 3 d (n = 4 replicates; error bars represent SD). Representative data from one of two independent experiments; (d) Bliss synergy plots for EW8 (left), RDES (center), and TC71 (right) cells treated with various combinations of GNF-7 and CPT for 5 d. Positive scores (red) indicate regions of synergy. Representative data from one of two independent experiments.; Figure S3: GNF-7 induces cell cycle arrest and reverses ES-related transcriptional signatures. (a) Histograms depicting cell cycle distribution based on the amount of PI fluorescent signal of EW8 and TOP1 KD cells treated with DMSO, 4 nM CPT or 40 nM GNF-7 for 24 h; (b) Cell cycle distribution of EW8 (left) and TOP1 KD (right) cells treated with either DMSO, 4 nM CPT, or 40 nM GNF-7 for 48 h (top) or 72 h (bottom). Numbers within the bars represent the percentage of cells in that phase; (c) Percentage of Fucci reporter-expressing EW8 cells fluorescing either red, green or both (yellow) at 30 min intervals after treatment with DMSO, 4 nM CPT or 40 nM GNF-7 (top panels). Example images of cells taken after 72 h of treatment (bottom panels). Scale bar, 400 μm. Representative data from one of two independent experiments (a-c); (d) GSEA enrichment plots for EW8 and TOP1 KD transcriptional profiles after 16 h of treatment with 40 nM GNF-7 when analyzed against the Kinsey: Targets of EWSR1/FLI1 Fusion UP gene set.; Figure S4: GNF-7 is a multi-kinase inhibitor. (a) Kinase trees mapping in situ kinase inhibition data from EW8 (left) and TOP1 KD (center) cells treated with 40 nM GNF-7 for 24 h. Circle color and size indicate percent inhibition. Kinase tree illustrations reproduced courtesy of Cell Signaling Technology, Inc.; (b) Table displaying all kinases in (a) inhibited > 50% in either cell line, with the actual percent inhibition values for both cell lines, as well as the difference in inhibition between the two lines; (c) Heat map of the percentage of viable EW8 or TOP1 KD cells 72 h after transfection with siRNAs targeting the indicated transcript, ranked by largest reduction in viability in the EW8 cells. Each data point represents the average value of three individual siRNAs.
