# Supplementary material for: The Kinase Inhibitor GNF-7 Is Synthetically Lethal in Topoisomerase 1-Deficient Ewing Sarcoma
Source: Cancers (Basel). 2025 Jul 26;17(15):2475. doi: 10.3390/cancers17152475 (PMC12346386; doi:10.3390/cancers17152475)
Supplement: Supplementary file 1 [file cancers-17-02475-s001.zip › cancers-3722875-supplementary.pdf]

Supplemental Figure S1

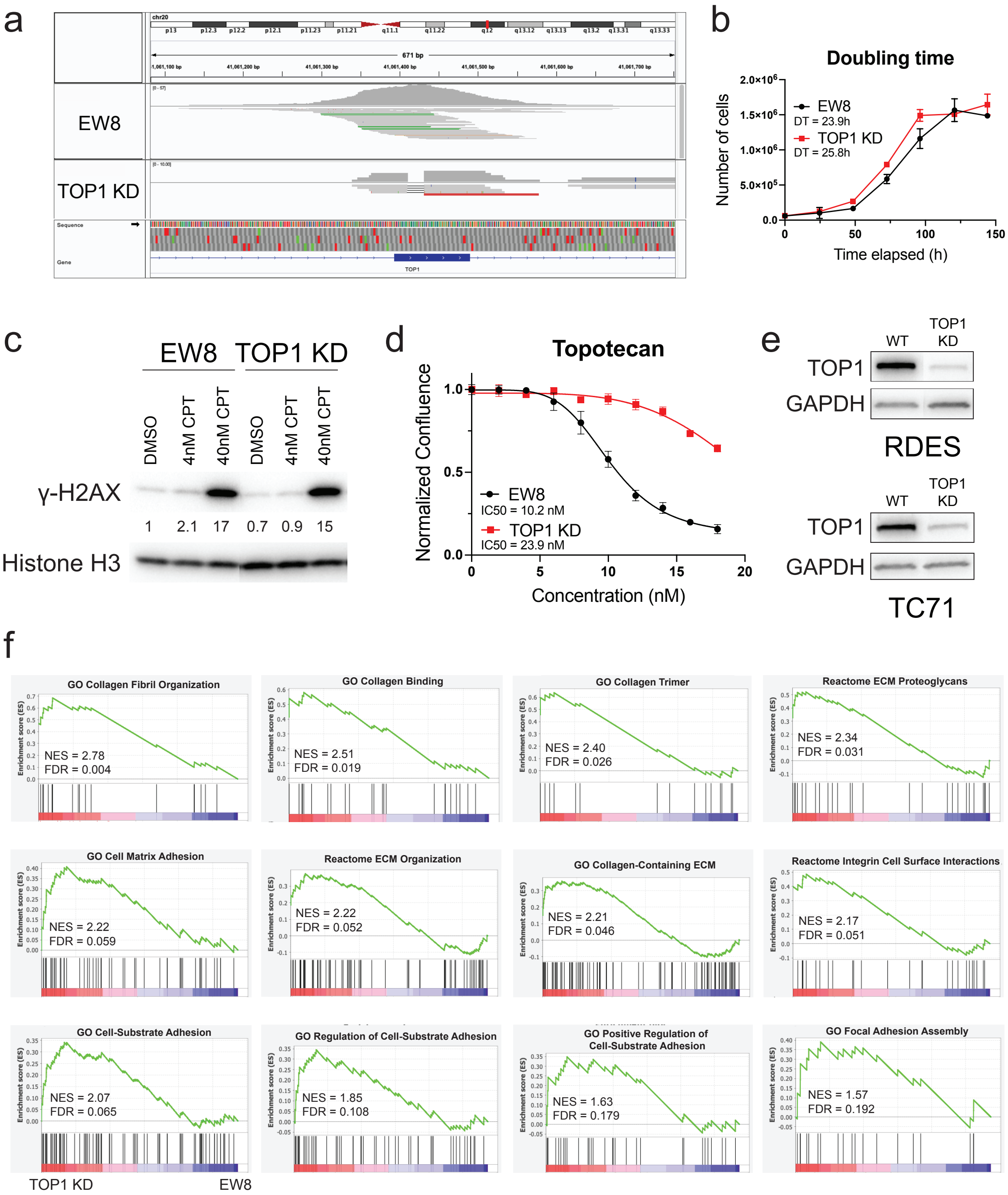

Supplementary Figure S1. TOP1 KD sensitizes ES cells to TOP1 inhibitors. **(a)** IGV figure showing exome sequencing reads over exon 3 of the TOP1 gene in parental EW8 and EW8 TOP1 KD cells; **(b)** Cell proliferation curves of untreated EW8 and TOP1 KD cells over 6 d (n = 2). Doubling time was calculated between days 3 and 4; **(c)** Western blot of γ-H2AX signal in EW8 and EW8 TOP1 KD cells treated with various doses of CPT for 24h; **(d)** Dose response curves for EW8 and TOP1 KD cells treated with various doses of topotecan for 3 d (n = 4 replicates; error bars represent SD); **(e)** Western blots of TOP1 expression in RDES and RDES TOP1 KD cells (top) or in TC71 and TC71 KD cells (bottom). Representative data from one of two independent experiments (**b-e**); **(f)** GSEA plots of enriched gene sets in untreated TOP1 KD vs. EW8 cells. Plots correspond to the gene sets shown in Fig. 1(g).

Supplemental Figure S2

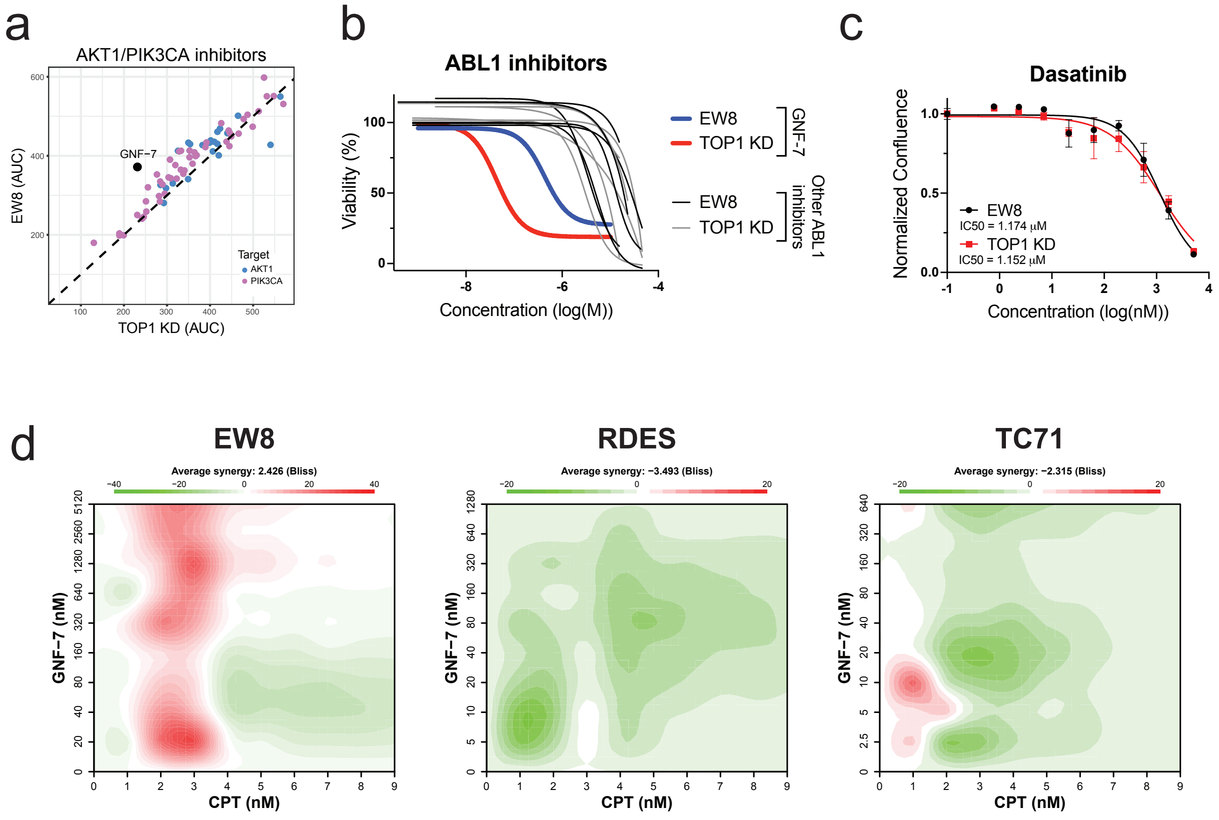

Supplementary Figure S2. Pharmacological characterization of EW8 and TOP1 KD cells. **(a)** Scatterplot of area under the dose response curves (AUC) for EW8 and TOP1 KD cells, highlighting AKT and PIK3CA inhibitors. The GNF-7 data point is shown for reference. Dashed line at y = x; **(b)** Dose response curves for EW8 and TOP1 KD cells from the MIPE compound library screen for select drugs that target ABL1; **(c)** Dose response curves for EW8 and TOP1 KD cells treated with various doses of dasatinib for 3 d (n = 4 replicates; error bars represent SD). Representative data from one of two independent experiments; **(d)** Bliss synergy plots for EW8 (left), RDES (center), and TC71 (right) cells treated with various combinations of GNF-7 and CPT for 5 d. Positive scores

(red) indicate regions of synergy. Representative data from one of two independent experiments.

Supplemental Figure S3

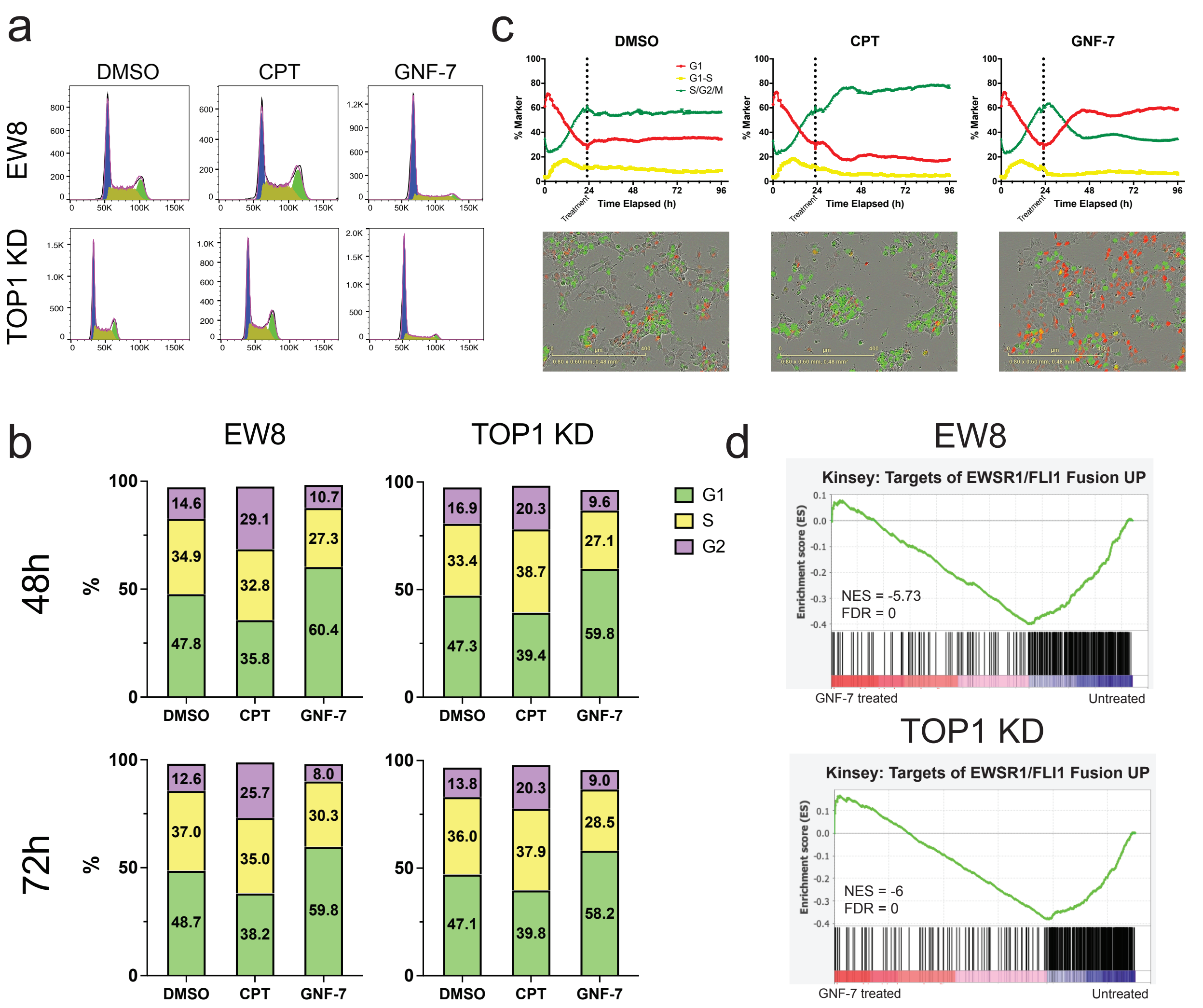

Supplementary Figure S3. GNF-7 induces cell cycle arrest and reverses ES-related transcriptional signatures. **(a)** Histograms depicting cell cycle distribution based on the amount of PI fluorescent signal of EW8 and TOP1 KD cells treated with DMSO, 4 nM CPT or 40 nM GNF-7 for 24 h; **(b)** Cell cycle distribution of EW8 (left) and TOP1 KD (right)

cells treated with either DMSO, 4 nM CPT, or 40 nM GNF-7 for 48 h (top) or 72 h (bottom). Numbers within the bars represent the percentage of cells in that phase; **(c)** Percentage of Fucci reporter-expressing EW8 cells fluorescing either red, green or both (yellow) at 30 min intervals after treatment with DMSO, 4 nM CPT or 40 nM GNF-7 (top panels). Example images of cells taken after 72 h of treatment (bottom panels). Scale bar, 400 µm. Representative data from one of two independent experiments **(a-c)**; **(d)** GSEA enrichment plots for EW8 and TOP1 KD transcriptional profiles after 16 h of treatment with 40 nM GNF-7 when analyzed against the Kinsey: Targets of EWSR1/FLI1 Fusion UP gene set.

Supplemental Figure S4

a

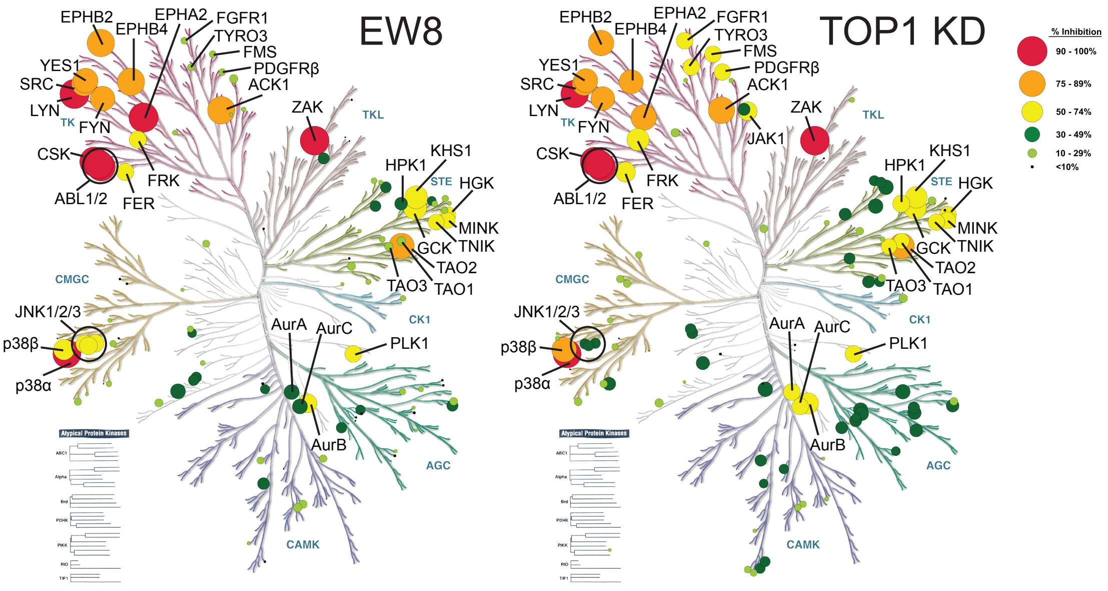

Illustration reproduced courtesy of Cell Signaling Technology, Inc. (www.cellsignal.com).

b

Kinase	% Inhibition (EW8)	% Inhibition (TOP1 KD)	Difference (TOP1 KD - EW8)
CSK	95.4	93.4	-2.0
EphA2	95.3	82.8	-12.5
LYN	94.7	89.6	-5.1
ZAK	94.0	93.8	-0.1
ABL1/2	91.5	92.0	0.5
p38α	91.3	91.1	-0.2
EphB2	89.3	82.8	-6.5
EphB4	89.2	78.2	-11.0
ACK1	88.8	84.9	-3.8
TAO2	82.7	81.8	-0.9
FYN	79.7	77.2	-2.4
SRC	79.7	77.2	-2.4
YES1	79.7	77.2	-2.4
GCK	75.0	71.5	-3.4
KHS1	74.2	70.4	-3.8
p38β	67.4	79.8	12.5
AurB	60.9	69.1	8.2
JNK1/2/3	57.8	33.8	-24.0
FER	56.0	65.1	9.1
FRK	55.9	71.9	15.9
PLK1	55.5	54.9	-0.6
HGK	51.8	54.1	2.3
MINK	51.8	54.1	2.3
TNIK	51.8	54.1	2.3
AurC	49.5	54.0	4.5
AurA	47.1	55.2	8.1
HPK1	41.9	55.5	13.7
TAO1/3	28.4	50.7	22.3
FGFR1	23.2	53.6	30.3
FMS	23.2	53.6	30.3
PDGFRβ	23.2	53.6	30.3
TYRO3	23.2	53.6	30.3
JAK1	21.1	61.4	40.3

c

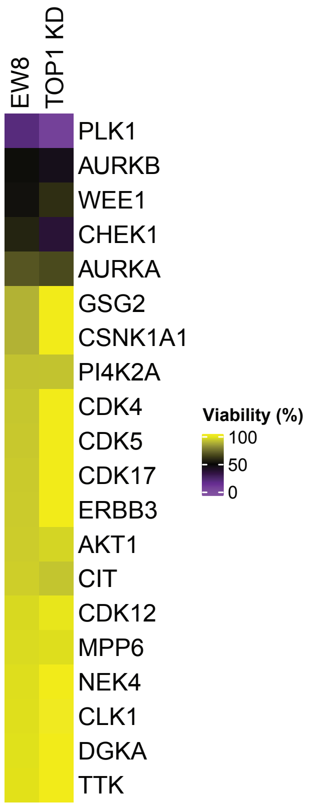

Supplementary Figure S4. GNF-7 is a multi-kinase inhibitor. **(a)** Kinase trees mapping *in situ* kinase inhibition data from EW8 (left) and TOP1 KD (center) cells treated with 40 nM GNF-7 for 24 h. Circle color and size indicate percent inhibition. Kinase tree illustrations reproduced courtesy of Cell Signaling Technology, Inc.; **(b)** Table displaying all kinases in **(a)** inhibited > 50% in either cell line, with the actual percent inhibition values for both cell lines, as well as the difference in inhibition between the two lines; **(c)** Heat map of the percentage of viable EW8 or TOP1 KD cells 72 h after transfection with siRNAs targeting the indicated transcript, ranked by largest reduction in viability in the EW8 cells. Each data point represents the average value of three individual siRNAs.
